# Reply to “How to consider the indication of implantable cardioverter defibrillator in the elderly patients”

**DOI:** 10.1002/clc.24208

**Published:** 2024-12-13

**Authors:** Michael Gotzmann, Marie Lewenhardt, Fabienne Kreimer

**Affiliations:** ^1^ Department of Cardiology and Rhythmology, St. Josef‐Hospital Ruhr University Bochum Bochum Germany

We would like to thank Naoya Kataoka and Teruhiko Imamura for their comments on our publication. While we strongly agree with some of their comments, we would like to take the opportunity to point out some aspects of our publication that may not have been described clearly enough.

We would like to clarify that in our study not only deceased patients with previous implantable cardioverter defibrillator (ICD) therapy were defined as patients with “benefit of ICD implantation,” but also patients with adequate ICD therapy who did not die during the observation period. In fact, of the 89 patients who received adequate ICD therapy, only 21 patients died during the study period.^1^ We would also like to point out that the investigation period of 4.2 years on average was not short. We therefore do not believe that an extended investigation period would have changed the results of the analysis.

In principle, however, we agree with Naoya Kataoka and Teruhiko Imamura on several points. The surgical risk and the risk of infection play a significant role in the risk‐benefit assessment of device therapy. In our study, however, we limited ourselves to a few endpoints and focused in particular on the major endpoint “death from any cause.”

Another important aspect is that comorbidities have a significant impact on the benefit of ICD therapy. A younger multimorbid patient may have a worse prognosis than an older patient. Nonetheless, we believe that considering age when deciding whether ICD therapy is appropriate can be a very simple but important contribution. As the benefits of ICD therapy in older patients have been repeatedly questioned in the past, this is particularly important.^2^, ^3^, ^4^ At the same time, in an increasingly aging society, a considerable proportion of old and very old patients are treated with an ICD. In Germany and the United States, the proportion of patients over 80 years of age who receive an ICD for primary prophylactic indications is approximately 14%, without convincing data being available. Our study revealed that the benefit of ICD therapy in this patient group remains to be critically assessed.

In other words, comorbidities are undeniably important factors for the potential benefit of ICD therapy, but also age, so taking comorbidities into account could help to make a benefit‐risk assessment before ICD implantation.

## Data Availability

Data are available on request due to privacy/ethical restrictions. The data that support the findings of this study are available on request from the corresponding author. The data are not publicly available due to privacy or ethical restrictions.
